# Rheumatoid factors do not predict cardiovascular disease and mortality in the general population in the Busselton Health Survey

**DOI:** 10.1186/s12891-017-1598-x

**Published:** 2017-05-26

**Authors:** Johannes Nossent, Warren Raymond, Mark Divitini, Matthew Knuiman

**Affiliations:** 1 School of Medicine & Pharmacology, The University of Western Australia, 35 Stirling Hwy (M503), Crawley, WA 6009 Australia; 2 Department of Rheumatology, Sir Charles Gairdner Hospital, Perth, WA Australia; 3 School of Population Health, The University of Western Australia, Perth, WA Australia; 4 Busselton Population Medical Research Institute, Busselton, WA Australia

**Keywords:** Rheumatoid factors, Cardiovascular disease, Mortality, Rheumatoid arthritis

## Abstract

**Background:**

Rheumatoid Factors (RF) are antibodies directed against the Fc portion of IgG and are involved in clearance of immune complexes. While RF can develop in a wide range of conditions, higher RF levels indicate a greater risk for a severe disease course in Rheumatoid Arthritis (RA) patients including cardiovascular complications and premature death. We investigated whether RF also constitute a risk factor for these outcomes in the general population.

**Methods:**

We included 2,323 participants (46% male, mean age 50 years) free of CVD at baseline in 1972. RF positivity was defined as a score of ≥2 by latex agglutination (scale 0–5). All outcomes during 42-year follow-up were obtained from state-wide registries. The predictive value of RF for coronary heart disease, all cardiovascular disease and all-cause mortality was estimated by adjusted hazard ratios (HR) from Cox regression models.

**Results:**

After adjustment for standard risk factors, RF positivity was not predictive of future CHD (HR 1.05, *p* = 0.61), CVD (HR 1,04, *p* = 0.63) or mortality (HR 1.03, *p* = 0.70) in the full CVD-free cohort. In an interaction model, RF in 41 out of 355 participants with an RA history was not predictive of CHD (HR 0.92, *p* = 0.77) or CVD events (HR 1.15, *p* = 0.51), but there was a borderline significant association with overall mortality (HR 1.41, CI 0.97–2.04, *p* = 0.07).

**Conclusions:**

RF detected by Latex agglutination do not independently predict future CHD, CVD or death in the general population. However, the presence of RF in the context of a history of RA is associated with a moderate, borderline significant increase in the long term adjusted risk for all-cause mortality.

**Electronic supplementary material:**

The online version of this article (doi:10.1186/s12891-017-1598-x) contains supplementary material, which is available to authorized users.

## Background

Rheumatoid Factors (RF) are a family of polyclonal antibodies directed against the Fc portion of IgG [1]. The functions of RF include immune complex (IC) clearance, complement fixation and antigen uptake by B cells for T cell presentation. The origin of RF is not well understood, but they can be induced bacterial lipopolysaccharides and Epstein-Barr virus [2–5]. The overall evidence points to RF development as part of normal host defense mechanism that involves an immune response to modified IgG. Consequently, RF are prevalent in a range of inflammatory conditions including Rheumatoid Arthritis (RA), where RF presence increases the risk for bony joint erosions, accelerated atherosclerosis, cardiovascular disease (CVD) and early mortality [6, 7]. Despite its inherent low specificity, RF testing is widely used as a screening test for rheumatic disease. It is unclear whether the incidental presence of RF in non-RA individuals reflects a state of chronic inflammation, which is a recognized risk factor for atherosclerosis [8]. RF as detected by Waaler-Rose assay was a risk factor for future coronary heart disease (CHD) as well as overall mortality in the general population of Iceland [9]. We investigated whether RF as detected by a routine Latex agglutination assay also indicate an increased risk for CHD, CVD and death in a community based cohort of Australian adults with long-term follow-up.

## Methods

### Study design and participants

Cross-sectional surveys were conducted in the district of Busselton in Western Australia (WA) every 3 years from 1966 to 1981 [10]. For this study the cohort was all adults who attended the 1972 survey and who were also tested for RF in the 1969 survey. Over 95% of the cohort were of Caucasian background. All participants gave informed consent and the surveys and this analysis were approved by the The Human Research Ethics Committee of the Department of Health of WA.

### Baseline measurements and follow-up outcome events

All baseline data for this cohort of participants was taken from the 1972 survey except for RF status which came from serum collected in the 1969 survey. In the 1972 survey, participants completed a baseline health and lifestyle questionnaire on smoking, diabetes, anti-hypertensive treatment, doctor-diagnosed RA, angina pectoris and myocardial infarction. Those with a history of CHD (based on Rose questionnaire and ECG) or stroke were excluded [10]. Blood pressure was measured by a mercury sphygmomanometer after five minutes rest in a sitting position. Serum collected in the 1969 survey was assayed for RF by latex-agglutination (Hyland, USA) with results semi quantitatively graded from 0 (negative) to 5 (strongly positive) with all scores ≥2 considered positive [11]. Outcome events over the period from 1973 to mid-2014 were ascertained from linked hospital admission records in the Western Australian Hospital Morbidity Data System (HMDS) and death records in the Western Australian Death Register. The HMDS is over 70% complete for the period 1973–1979 (not all hospitals provided data) and over 99% complete for 1980 to 2014 [12]. ICD-9 codes were used to identify relevant events 1973–1999 and ICD-10 codes for 2000–2014. Outcomes were time to first CHD event, time to first CVD event (hospital admission for CHD, stroke, congestive heart failure, peripheral arterial disease or death from cardiovascular disease) and time to death from any cause. As HMDS was incomplete during the period 1973–1979 we also recorded new doctor-diagnosed CHD and CVD events reported by participants in surveys during that period. This increased the number of participants with a CHD event from 685 to 775 (i.e gain of 90) and the number of participants with a CVD event from 1157 to 121 (i.e gain of 55).

### Statistical analysis

Results presented are for the cohort with no history of CHD or stroke at baseline in 1972. Cox regression models for time from baseline to first outcome event were used to obtain adjusted hazard ratios (with 95% confidence intervals) for history of RA (yes, no) and for RF considered both as a continuous score (values 0 to 5) to provide the trend test *p*-value and also as a binary variable (negative 0–1, positive 2–5). We formed the RF groups of 0–1 and 2–5 because the distribution of RF score was uneven with 1778 people with score 0, 190 with score 1, 182 with score 2, 118 with score 3, 54 with score 4 and 1 with score 5 and the number of outcome events was less than 100 for many of the groups from 1 to 5. Two models were fitted, the first adjusted for age and sex only (Additional file 1: Tables S1 and S2), and the second adjusted for age, sex, smoking, BMI, SBP, hypertension medication, cholesterol, and diabetes (Tables 2 and 3). Results from Cox regression models are presented as estimated hazard ratios (with 95% confidence interval and *p*-value). The proportional hazards assumptions for the effects of RF and RA on outcomes were tested via an interaction with follow-up time and in all cases the assumption was not violated (with interaction *p*-values ranging from 0.318 to 0.859).

## Results

The study cohort included 2323 participants free of CHD or stroke at baseline. RF prevalence was 15.3% at the chosen cut-off level of ≥2. Table 1 shows the descriptive statistics for the cohort by RF status, RA status and overall. The cohort has a behavioural and biomedical risk factor profile typical of the general population at that time [10].

**Table 1:** Descriptive statistics for the cohort free of CHD and stroke at baseline in 1972. Table shows mean (SD), percent or *N* (%)

	RF		RA		
	0–1	2–5	No	Yes	Total
Characteristic or measure	(*n* = 1968)	(*n* = 355)	(*n* = 2124)	(*n* = 199)	(*n* = 2323)
Male (%)	48.9	31.5	47.0	38.2	46.3
Age (years)	49.2 (14.4)	55.6 (14.2)	49.4 (14.6)	57.8 (12.5)	50.1 (14.6)
Smoking					
Never (%)	47.5	53.0	48.1	51.3	48.3
Ex (%)	22.9	21.4	22.7	21.6	22.6
Current (%)	29.6	25.6	29.2	27.1	29.0
BMI (kg/m^2^)	25.1 (3.6)	25.1 (3.6)	25.1 (3.6)	25.6 (4.2)	25.1 (3.6)
Systolic BP (mm Hg)	136 (20)	139 (22)	135 (20)	143 (23)	136 (21)
Diastolic BP (mm Hg)	78 (13)	80 (12)	78 (13)	81 (12)	79 (13)
Hypertension medication (%)	6.6	11.5	6.9	12.6	7.4
Cholesterol (mmol/L)	6.33 (1.24)	6.58 (1.37)	6.34 (1.24)	6.58 (1.46)	6.36 (1.27)
Diabetes mellitus (%)	1.7	2.3	1.6	4.0	1.8
History of Rheumatoid Arthritis (%)	8.0	11.5	-	-	8.6
Rheumatoid Factor					
Score (0–5)	0.10 (0.30)	2.65 (0.74)	0.47 (0.98)	0.67 (1.18)	0.49 (1.00)
Positive (2–5) (%)	-	-	14.8	20.6	15.3
Follow-up time (years)	26.1 (13.9)	22.1 (14.0)	26.0 (14.0)	20.0 (13.2)	25.5 (14.0)
No. with CHD event	647 (32.9)	128 (36.1)	699 (32.9)	76 (38.2)	775 (33.4)
No. with CVD event	1005 (51.1)	207 (58.3)	1089 (51.3)	123 (61.8)	1212 (52.2)
No. of deaths	1141 (58.0)	241 (67.9)	1229 (57.9)	153 (76.9)	1382 (59.5)

As results from the model that adjusted for age and sex only (Additional file 1: Tables S1 and S2) were essentially the same as the results from the fully adjusted model (Tables 2 and 3), only results from the fully adjusted models are described here. The presence of RF or a history of RA, when considered separately or together (i.e. adjusted for the other) were not predictive of CHD or CVD events (all *p*-values > 0.3) (Table 2). A positive RF was associated with a 5% increased risk of CHD events (HR 1.05, 95% CI 0.87–1.28, *p* = 0.61) and a 4% increased risk of CVD events (HR 1.04, 95% CI 0.89–1.21, *p* = 0.63). A doctor diagnosis of RA was associated with a 1% increased risk of CHD events (HR 1.01, 95% CI 0.79–1.28, *p* = 0.94) and CVD events (HR 1.01, 95% CI 0.83–1.22, *p* = 0.95). Similarly, the presence of RF or a history of RA were not associated with all-cause mortality (all *p*-values >0.5) (Table 3). A positive RF was associated with a 3% increased risk of death (HR 1.03, 95% CI 0.89–1.19, *p* = 0.70) and a doctor diagnosis of RA was associated with a 0% increased risk of death (HR 1.00, 95% CI 0.84–1.19, *p* = 0.99). Increasing the cut-off level for RF positivity to an agglutination score ≥ 3 or ≥4 decreased the prevalence of RF to 7.4 and 2.2%, but did not change any of the follow-up results markedly.

**Table 2:** Estimated adjusted hazard ratios for rheumatoid factor (RF) and rheumatoid arthritis (RA) in relation to CHD and CVD events in the cohort free of CHD and stroke at baseline in 1972. Table shows hazard ratio, 95% CI and *p*-value

	CHD		CVD	
Risk factor	HR^a^ (95% CI)	*p*-value	HR^a^ (95% CI)	*p*-value
RA (not adjusted for RF)	1.01 (0.79, 1.28)	0.943	1.01 (0.83, 1.22)	0.950
RA (adjusted for RF)	1.01 (0.79, 1.28)	0.951	1.01 (0.83, 1.21)	0.953
RF score^b^ (not adjusted for RA)	1.02 (0.95, 1.10)	0.522	1.03 (0.97, 1.09)	0.352
RF score^b^ (adjusted for RA)	1.02 (0.95, 1.10)	0.523	1.03 (0.97, 1.09)	0.352
RF positive (not adjusted for RA)	1.05 (0.87, 1.28)	0.610	1.04 (0.89, 1.21)	0.633
RF positive (adjusted for RA)	1.05 (0.87, 1.28)	0.611	1.04 (0.89, 1.21)	0.633
RF positive (for RA = no)^c^	1.07 (0.87, 1.31)	0.516	1.02 (0.87, 1.20)	0.787
RF positive (for RA = yes)^c^	0.92 (0.51, 1.64)	0.770	1.15 (0.75, 1.77)	0.512

^a^From Cox model adjusted for age, sex, smoking, BMI, SBP, hypertension medication, cholesterol and diabetes

^b^HR is for an increase of one in the score

^c^From Cox model that included interaction between RF and RA

**Table 3:** Estimated adjusted hazard ratios for rheumatoid factor (RF) and rheumatoid arthritis (RA) in relation to Death (any cause) in the cohort free of CHD and stroke at baseline in 1972. Table shows hazard ratio, 95% CI and *p*-value

	Death	
Risk factor	HR^a^ (95% CI)	*p*-value
RA (not adjusted for RF)	1.00 (0.84, 1.18)	0.994
RA (adjusted for RF)	1.00 (0.84, 1.18)	0.995
RF score^b^ (not adjusted for RA)	1.01 (0.96, 1.07)	0.590
RF score^b^ (adjusted for RA)	1.01 (0.96, 1.07)	0.590
RF positive (not adjusted for RA)	1.03 (0.89, 1.19)	0.698
RF positive (adjusted for RA)	1.03 (0.89, 1.19)	0.698
RF positive (for RA = no)^c^	0.98 (0.84, 1.14)	0.785
RF positive (for RA = yes)^c^	1.41 (0.97, 2.03)	0.069

^a^From Cox model adjusted for age, sex, smoking, BMI, SBP, hypertension medication, cholesterol and diabetes

^b^HR is for an increase of one in the score

^c^From Cox model that included interaction between RF and RA

Models that included an interaction between RF and history of RA showed that the effect of RF on CHD events (interaction *p* = 0.62) and CVD events (interaction *p* = 0.61) was not significantly different in people with and without a history of RA (Table 2). In people with a history of RA, a positive RF was associated with an 8% decreased risk of CHD events (HR 0.92, 95% CI 0.51–1.64, *p* = 0.77) and a 15% increased risk of CVD events (HR 1.15, 95% CI 0.75–1.77, *p* = 0.51). However, in relation to all-cause mortality, the interaction between RF and history of RA approached significance (*p* = 0.075). In people with a history of RA, a positive RF was associated with a 41% increased risk of death (HR 1.41, 95% CI 0.97–2.04, *p* = 0.069).

## Discussion

This population based cohort study with long-term follow-up found a significant population prevalence of RF as detected by latex agglutination, but no evidence that RF or having a history of RA in itself were independent risk factors for CHD, CVD events or all-cause mortality. Only the presence of RF in patients with a history of RA was associated with an increase in long term mortality risk.

Using moderately strict cut-off levels for RF positivity, we found a high prevalence of RF at 15% in this Western Australian population. Increasing cut-off levels understandably reduced the prevalence of RF to 7.4% (at cut-off ≥3) and 2.2% (at cut-off ≥4), but despite the presumed increase in specificity, the use of different cut-off levels did not significantly change the overall risks for CVD or death. Our results are largely in agreement with findings in an earlier population study performed in Iceland [9], where RF as measured by latex agglutination was present in almost 11% of the participants, but had no predictive value for coronary heart disease or death during a median follow-up of 23 years. Interestingly, in the Icelandic study a subgroup of latex RF positive individuals tested positive (titre >1:10) by the Waaler–Rose (WR) erythrocyte agglutination assay, which is an assay with significantly higher specificity for RA. Even though the assays in the two studies are not directly comparable, the WR positive patients in the Icelandic study form a realistic approximation of our RA cohort. Under this assumption, the fully adjusted HR for overall mortality of 1.40 (1.14 to 1.72) for WR positive participants in the Icelandic study fits well with the HR of 1.41 (0.97–2.03) for participants reporting RA in this cohort (Table 3).

While the exact biological properties of RF that lead to the associated risks for joint erosions and accelerated atherosclerosis in RA patients are not well defined, this could involve affinity maturation of the RF response, which under the influences of of endo- and exogenous triggers occurs through accumulated somatic mutations in RF producing autoreactive B cells clones [13, 14]. The more stringent conditions in the Waaler-Rose assay capture this underlying process better than the Latex agglutination for RF detection. The limitations of this study include the use of patient reported, but doctor-diagnosed RA at a time in history where there was limited clinical experience with RF testing. Also, there is a possibility that RF effects are too small for this study to detect. A power calculation showed a 90% power to detect a HR of 1.32 for RF (positive vs. negative) in relation to CHD events and a HR of 1.25 in relation to CVD events. The long follow up period, the ability to use different RF cut-off levels and to fully adjust for traditional risk factors as well the completeness of outcome capturing provide considerable strength to this study.

## Conclusions

RF as detected by Latex agglutination do not independently predict cardiovascular disease or death in the general population. These data provide reassurance for physicians faced with a false positive RF test detected by latex agglutination.

## Additional file

Additional file 1: Additional data on RF as predictor of CHD, CVD and death. Tables showing the estimated adjusted hazard ratios for rheumatoid factor (RF) and rheumatoid arthritis (RA) presence in the cohort free of CHD and stroke at baseline in 1972 in relation to: Subsequent CHD and CVD events (Table S1), Subsequent death (any cause) (Table S2). (DOCX 17 kb)

## Abbreviations

BHS: Busselton health survey
BMI: Body mass index
CI: Confidence interval
CRP: C-reactive protein
CVD: cardiovascular disease
CVE: cardiovascular events
HMDS: Western Australian hospital morbidity data collection
HR: Hazard ratio
IC: Immune complex
ICD: International classification of diseases
RA: Rheumatoid arthritis
RF: Rheumatoid factors
SBP: Systolic blood pressure
WA: Western Australia
