# Supplementary material for: Rheumatoid factors do not predict cardiovascular disease and mortality in the general population in the Busselton Health Survey
Source: BMC Musculoskelet Disord. 2017 May 26;18:221. doi: 10.1186/s12891-017-1598-x (PMC5446675; doi:10.1186/s12891-017-1598-x)
Supplement: Additional file 1: — Additional data on RF as predictor of CHD, CVD and death. Tables showing the estimated adjusted hazard ratios for rheumatoid factor (RF) and rheumatoid arthritis (RA) presence in the cohort free of CHD and stroke at baseline in 1972 in relation to: Subsequent CHD and CVD events (Table S1), Subsequent death (any cause) (Table S2). (DOCX 17 kb) [file 12891_2017_1598_MOESM1_ESM.docx]

**Additional file 1**

Table S1. Estimated adjusted hazard ratios for rheumatoid factor (RF) and rheumatoid arthritis (RA) in relation to CHD and CVD events in the cohort free of CHD and stroke at baseline in 1972. Table shows hazard ratio, 95% CI and p-value.

|  | CHD | | CVD | |
| --- | --- | --- | --- | --- |
| Risk factor | HR^1^ (95% CI) | p-value | HR^1^ (95% CI) | p-value |
| RA (not adjusted for RF) | 1.02 (0.81, 1.30) | 0.842 | 1.01 (0.83, 1.22) | 0.932 |
| RA (adjusted for RF) | 1.02 (0.81, 1.30) | 0.848 | 1.01 (0.83, 1.22) | 0.932 |
| RF score^2^ (not adjusted for RA) | 1.02 (0.95, 1.10) | 0.520 | 1.02 (0.97, 1.08) | 0.378 |
| RF score^2^ (adjusted for RA) | 1.02 (0.95, 1.10) | 0.521 | 1.02 (0.97, 1.08) | 0.378 |
| RF positive (not adjusted for RA) | 1.05 (0.86, 1.27) | 0.635 | 1.03 (0.88, 1.20) | 0.715 |
| RF positive (adjusted for RA) | 1.05 (0.86, 1.27) | 0.637 | 1.03 (0.88, 1.20) | 0.716 |
| RF positive (for RA = no )^3^ | 1.07 (0.87, 1.31) | 0.516 | 1.02 (0.86, 1.20) | 0.828 |
| RF positive (for RA = yes )^3^ | 0.89 (0.50, 1.59) | 0.693 | 1.11 (0.72, 1.70) | 0.642 |

^1^ From Cox model adjusted for age and sex.
^2^ HR is for an increase of one in the score
^3^ From Cox model that included interaction between RF and RA

Table S2. Estimated adjusted hazard ratios for rheumatoid factor (RF) and rheumatoid arthritis (RA) in relation to Death (any cause) in the cohort free of CHD and stroke at baseline in 1972. Table shows hazard ratio, 95% CI and p-value.

|  | Death | |
| --- | --- | --- |
| Risk factor | HR^1^ (95% CI) | p-value |
| RA (not adjusted for RF) | 1.02 (0.86, 1.21) | 0.797 |
| RA (adjusted for RF) | 1.02 (0.86, 1.21) | 0.796 |
| RF score^2^ (not adjusted for RA) | 1.10 (0.96, 1.06) | 0.741 |
| RF score^2^ (adjusted for RA) | 1.10 (0.96, 1.06) | 0.740 |
| RF positive (not adjusted for RA) | 1.10 (0.87, 1.16) | 0.904 |
| RF positive (adjusted for RA) | 1.10 (0.87, 1.16) | 0.904 |
| RF positive (for RA = no )^3^ | 0.96 (0.82, 1.12) | 0.627 |
| RF positive (for RA = yes )^3^ | 1.36 (0.94, 1.96) | 0.105 |

^1^ From Cox model adjusted for age and sex.
^2^ HR is for an increase of one in the score
^3^ From Cox model that included interaction between RF and RA
